# Prefilled Cyclic Olefin Sterilized Syringes of Norepinephrine Injection Solution Do Not Need to Be Stabilized by Antioxidants

**DOI:** 10.1208/s12249-020-01784-z

**Published:** 2020-08-30

**Authors:** Karin H. M. Larmené- Beld, Stefan van Berkel, Rommert Wijnsma, Katja Taxis, Henderik W. Frijlink

**Affiliations:** 1 Department of Clinical Pharmacy, Isala, Dokter van Heesweg 2, 8025 AB Zwolle, The Netherlands; 2 Unit of PharmacoTherapy, -Epidemiology & PharmacoEconomics (PTE2), Groningen Research Institute of Pharmacy, University of Groningen, Groningen, The Netherlands; 3 Department of Pharmaceutical Technology and Biopharmacy, University of Groningen, Groningen, The Netherlands

**Keywords:** norepinephrine, stability, antioxidant, syringe

## Abstract

Norepinephrine is a potent α-sympathomimetic drug which plays an important role in the acute treatment of hypotension and shock. Commercially available norepinephrine solutions contain sodium metabisulfite (Na2S2O5) as an antioxidant. However, prefilled cyclic olefin polymer syringes are not compatible with sodium metabisulfite. The aim of this study was to develop a new formulation of 0.1-mg/mL norepinephrine solution without sodium metabisulfite which is chemically stable and sterile and can be stored in prefilled polymer syringes. Formulation studies were performed with 0.1-mg/mL norepinephrine solution with 0, 0.05, or 0.1% ascorbic acid added as antioxidant. The syringes were filled under nitrogen gassing, stored at 20 ± 5°C, and protected from daylight. Based on the formulation test results, the final formulation was defined and stability testing at 20 ± 5°C was performed measuring norepinephrine concentration, pH, clarity, color of the solution, subvisible particles, and sterility at time intervals up to 12 months. The norepinephrine concentrations at *t* = 22 weeks were 100.4%, 95.4%, and 92.2% for the formulations with no ascorbic acid and with 0.05% and 0.10% ascorbic acid, respectively. Three batches for the stability study were produced containing norepinephrine, sodium edetate, sodium chloride, and water for injections filled under nitrogen gassing and stored at 20 ± 5°C. Norepinephrine concentrations were respectively 98.8%, 98.6%, and 99.3% for batches 1, 2, and 3 at *t* = 12 months. It can be concluded that norepinephrine (0.1 mg/mL) solution without metabisulfite is stable for at least 12 months at room temperature when protected from daylight.

## INTRODUCTION

Norepinephrine is a potent α-sympathomimetic drug which plays an important role in the acute treatment of hypotension and shock. In most European countries, commercially available preparations contain 1 or 5 mg/mL norepinephrine supplied as vials. Usually, the injection is diluted with sodium chloride 0.9% before administration. Preparation and administration of parenteral medication in the hospital setting are associated with error rates up to 48% (1–3). Providing ready-to-administer medication prepared in the pharmacy department has been suggested frequently to reduce medication errors (4,5). This is implemented in hospitals across Europe (6,7). A new development in this area is ready-to-administer prefilled sterilized syringes (PFSS) produced by the hospital pharmacy. The use of PFSS products eliminates the preparation step in the ward, thereby preventing medication errors (4,8).

In The Netherlands, norepinephrine concentrations of 0.05, 0.1, 0.2, or 0.4 mg/mL are available as ready-to-use products (vials) but not as ready-to-administer syringes. Due to the catechol substructure, norepinephrine can easily undergo oxidation resulting in the formation of adrenolutines, adrenochromes, and oxadrenochromes (the typical black-colored insoluble particles). This process is catalyzed by light, oxygen, elevated temperatures, heavy metals, basic conditions, and several excipients (9). In order to prevent the degradation and discoloration, commercially available products are manufactured in brown glass vials, airtight sealing, blanketing with nitrogen, addition of metal chelators such as ethylenediamine tetraacetic acid (EDTA), and addition of antioxidants such as sulfites or ascorbic acid, and they are to be stored at low temperatures (10). For optimum stability, a pH range of 3.6–6.0 is recommended for the norepinephrine solution (10,11). All commercially available norepinephrine solutions contain sodium metabisulfite (Na_2_S_2_O_5_) as an antioxidant. However, the cyclic olefin polymer syringes used for delivering ready-to-administer syringes are not compatible with sodium metabisulfite due to brown colorization of the syringes during sterilization. A further disadvantage of sodium metabisulfite is the possible adverse events due to sulfite hypersensitivity of patients which can result in symptoms varying from mild discomfort up to life-threatening episodes and even death (12,13). In a recently published systematic review, various results have been found about the stability of epinephrine (14).

The purpose of this study was to develop a formulation of 0.1-mg/mL norepinephrine solution for use in prefilled syringes which is chemically stable and sterile and which is free of sodium metabisulfite.

## MATERIALS AND METHODS

### Materials

Norepinephrine tartrate monohydrate was obtained from Cambrex Profarmaco Milano Srl (Milan, Italy). The formulation contained the following excipients: sodium edetate (Akzo Nobel Functional Chemical, Arnhem, Netherlands) and sodium chloride (ESCO France SAS, Dombasle, France). Ascorbic acid was purchased from Celanese, Denmark.

The cyclic olefin polymer (COP) syringes used were the BD Sterifill Advance™ 50-mL syringes from Becton Dickinson (BD) Medical Pharmaceutical systems, with a Luer Lock Adaptor and screwed tip cap. Filled syringes were closed with a bromobutyl plunger stopper from Datwyler.

#### Preparation Test Solutions

Formulation tests were performed with 0.1-mg/mL norepinephrine solutions containing 0.1 mg/mL sodium edetate, 8 mg/mL sodium chloride, and water for injections. To this solution 0, 0.05, or 0.1% ascorbic acid was added as an antioxidant. The test solutions were prepared on a laboratory scale and filled under nitrogen gassing. The filled syringes were not sterilized. The syringes were stored at room temperature (20 ± 5°C), protected from daylight. The norepinephrine concentration was determined after storage for 0, 1, 3, 7, 13, and 22 weeks.

For stability testing, 3 batches were produced. The formulation contained norepinephrine 0.11 mg/mL, sodium edetate 0.1 mg/mL, sodium chloride 8 mg/mL in water for injections. An overage of 10% norepinephrine was used for batch 1 and 2 because historical data show a loss of norepinephrine during the sterilization process. For batch 3, the overage was reduced to 5% because the loss in the validation batches 1 and 2 was less than expected. See also Table I for the formulation composition for testing and stability batches. Acceptance criteria for release were 95–105%; for the stability study, these were 90–110% based on a declaration of norepinephrine of 0.1 mg/mL. The norepinephrine solution was produced in a 400-L stainless steel vessel. The pH of the bulk solution was set to 3.8–3.9 (with 10% HCl or 2-M NaOH solutions), and the oxygen level in the solution was reduced to < 1% by nitrogen gassing. The syringes were filled with a semi-automatic filling and closing machine, filling and stopper placement were performed under nitrogen gassing. The syringes were terminally sterilized for 15 min at 121°C. For stability testing, performed after storage at 20 ± 5°C, the following parameters were determined: norepinephrine concentration, pH, clarity, color of solution, subvisible particles, and sterility. The time intervals were according to ICH guidelines (16).

**Table I:** Formulation Composition of Norepinephrine Solution

	Norepinephrine Concentration	Antioxidant	Osmotic agent
Formulation tests			
Batch 1	0.1 mg/mL	0.1 mg/mL sodium edetate No ascorbic acid	8 mg/mL sodium chloride
Batch 2	0.1 mg/mL	0.1 mg/mL sodium edetate 0.05% ascorbic acid	8 mg/mL sodium chloride
Batch 3	0.1 mg/mL	0.1 mg/mL sodium edetate 0.1% ascorbic acid	8 mg/mL sodium chloride
Stability study			
Batch 1	0.110 mg/mL	0.1 mg/mL sodium edetate	8 mg/mL sodium chloride
Batch 2	0.110 mg/mL	0.1 mg/mL sodium edetate	8 mg/mL sodium chloride
Batch 3	0.105 mg/mL	0.1 mg/mL sodium edetate	8 mg/mL sodium chloride

### Analytical Method

#### Analysis of Norepinephrine

The UHPLC system Agilent 1290 Infinity (Agilent, Santa Clara, California, USA) comprised an Agilent G4220A Binary Pump, an Agilent G4226A Autosampler, an Agilent G1316C Column Compartment, and an Agilent G4212A Diode Array Detector. The Agilent OpenLab CDS (EZChrom edition) was used as a processing module to obtain the results of this study. The chromatographic separations were performed on a Kinetex Biphenyl column (Phenomenex, Torrance, California, USA) with the following dimensions: particle diameter 2.6 μm, size 3.0 × 100 mm. The mobile phase consists of a mixture of deionized water (Millipore, Burlington, Massachusetts, USA); acetonitrile, HPLC gradient grade (Boom, Meppel, The Netherlands); and trifluoroacetic acid, reagent grade (Merck, Darmstadt, Germany). The mobile phase consisted of an acidified (0.1% trifluoroacetic acid) deionized water (solution A) partly mixed with a solution of 20% acetonitrile and 80% deionized water (solution B). The ratio of the mobile phase was set at 98.0% solution A and 2.0% solution B with a flow of 0.4 mL/min to obtain the isocratic separation of the components in the formulated samples. The injection volume was set at 5 μl. The temperature of the autosampler and column compartment was set at 10°C and 30°C respectively. A washing fluid of 5% acetonitrile was used to avoid sample carryover. Peaks were detected at a wavelength of 280 nm. The performance has been monitored by the analysis of a system suitability solution which contained norepinephrine (50 mg/L) and epinephrine (25 mg/L). Parameters such as retention time, resolution, and symmetry factor verified the suitability of the system. The standard solution containing norepinephrine 50 mg/L was freshly prepared for each calibration curve. Samples were diluted with deionized water to obtain a final concentration of around 50 mg/L.

#### Validation of the Analytical Method

The stability-indicating nature of the analytical method was confirmed by subjecting norepinephrine to thermal, photolytic, oxidative, and hydrolytic stress conditions. Norepinephrine (0.1 mg/mL) solutions stressed by hydrogen peroxide 0.1%, hydrochloric acid 0.06 M, or sodium hydroxide 0.06 M were filled into BD Sterifill Advance™ 50-mL syringes. To test the thermal condition, stressed and reference solutions were stored at 80°C and analyzed for the norepinephrine concentration. The temperature of 80°C was chosen because this was the highest possible temperature to perform the test, and also because the expected degradation will occur at this temperature (10). Test solutions were analyzed conform to the UHPLC method described above, with no interfering peaks being detected, demonstrating the selectivity of the analytical method.

Further validation was performed to demonstrate linearity, accuracy, and precision of the method according to ICH guidelines (16). For the linearity, the calibration curve was prepared using the peak areas obtained from the chromatograms of the injections with a known concentration of norepinephrine in a range of 25.0 to 75.0 mg/L. The relationship of peak area to concentration was found to be linear throughout the studied concentration range. The accuracy was determined at 3 levels of norepinephrine (0.05 mg/mL, 0.10 mg/mL, 0.15 mg/mL). An average recovery between 97.0 and 103.0% was obtained. The determination of the intermediate precision was performed on different days, with varying materials and systems. The relative standard deviation of the repeatability of the method was ≤ 2.0%. The average pooled standard deviation of the intermediate precision was ≤ 5.0%.

### Acceptance Criteria Norepinephrine Solution

According to different monographs of the European Pharmacopoeia and ICH guideline Q6A, several tests were performed in the stability study. An overview of the performed tests is given in Table II. No tests on impurities and extractables were performed due to an extensive qualification program performed for the primary container (17), and the raw materials used in the production process. All active pharmaceutical ingredients (API) raw material are approved in advance by the manufacturer with a certificate of analysis complies with the Ph. Eur. 9.0 of the Noradrenalin tartrate monograph.

**Table II:** Acceptance Criteria Norepinephrine Solution

Test	Monograph	Description/acceptance limit
1. Clarity and degree of opalescence of the solution	Ph. Eur. 2.2.1.	The clarity of the solution is the same as that of *water R*. The absence of any particles or inhomogeneity’s in a solution results in a clear solution.
2. Degree of coloration of the solution	Ph. Eur. 2.2.2	Examination of the degree of coloration of the solution in the range brown-yellow-red; ≤ B9
3. pH of the solution	Ph. Eur. 2.2.3	3.6–6.0
4. Identity sodium	Ph. Eur 2.3.1	Positive
5. Concentration norepinephrine	UHPLC method	95–105% at release; 90–110% end of shelf life
6. Concentration chloride		Titration method with silver nitrate. Concentration at release 95–105% (4.61–5.10 mg/ml) Concentration end of shelf life 90–110% (4.37–5.34 mg/ml)
7. Subvisible particles	Ph. Eur. 2.9.19	According to method 1. Light obscuration particle count test: ≤ 6000 particles/syringe for particles ≥ 10 μm and ≤ 600 particles/syringe for particles ≥25 μm
8. Closure integrity test	Ph. Eur. 3.2.9./manipulated	A dye immersion test with 0.1% methylene blue. Immerse the syringes in a 1 g/L solution of methylene blue and reduce the external pressure by 27 kPa for 10 min. Restore atmospheric pressure and leave the vials immersed for 30 min. Rinse the outside of the syringes. None of the vials contains any trace of colored solution.
9. Sterility	Ph. Eur. 2.6.1	Sterile
10. Extractable volume	Ph. Eur. 2.9.17	The volume extracted is not less than the nominal volume.

## RESULTS

### Formulation Tests

**Fig. 1:**
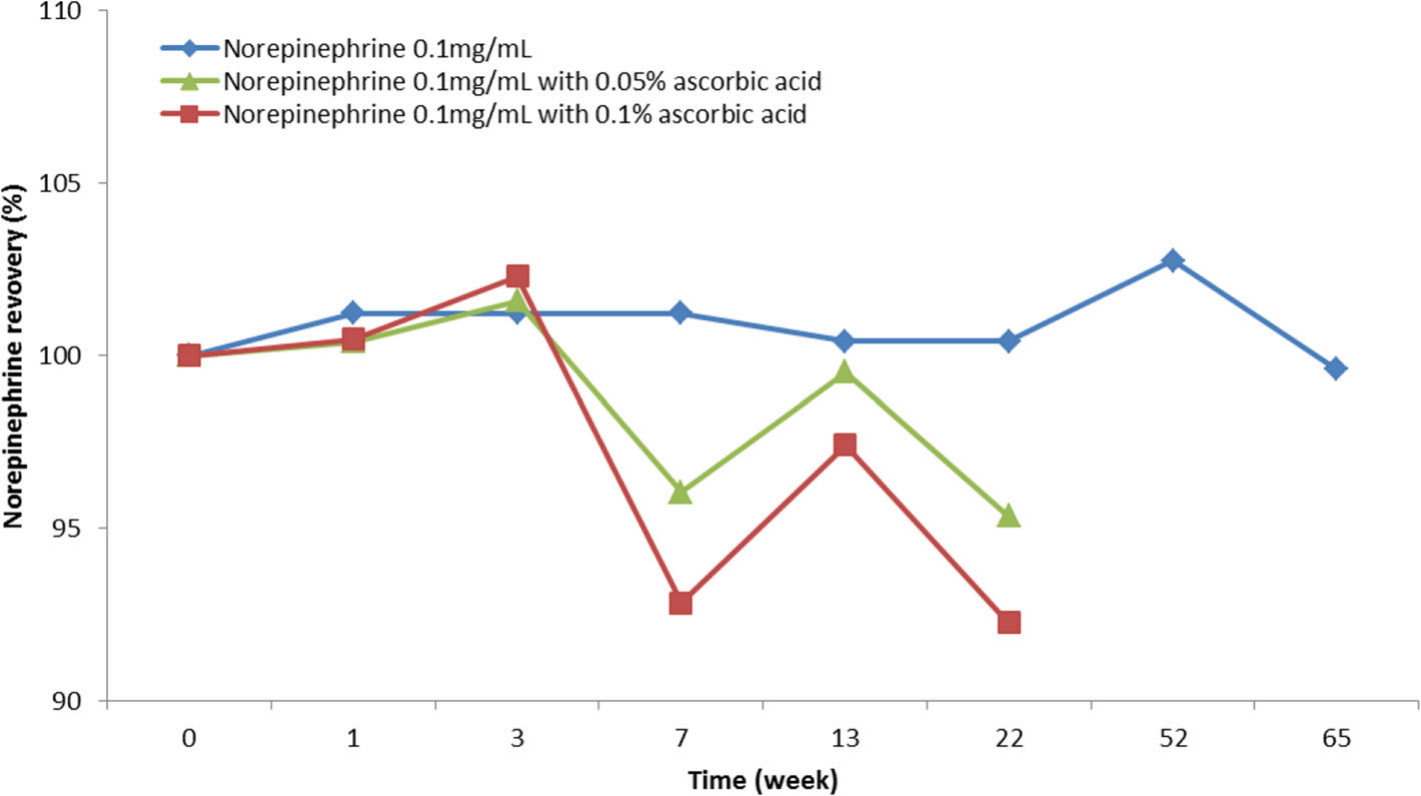
Norepinephrine recovery (%) in formulation study

The pH of the norepinephrine solution at *t* = 0 was 3.8, 3.3, and 3.1 for solutions without ascorbic acid or with 0.05% or 0.10% ascorbic acid, respectively. After storage for 5 months, norepinephrine levels in these solutions were 100.4%, 95.4%, and 92.2% (relative to the concentration of *t* = 0) respectively, as shown in Fig. 1. All results were within specification (90–110%) at *t* = 5 months with less variability in norepinephrine concentration during the study period when no antioxidant was added to the solution. Based on these results, it was decided to produce the batches for the stability studies without an antioxidant. The following formulation was produced: norepinephrine 0.11 mg/mL, sodium edetate 0.1 mg/mL, sodium chloride 8 mg/L with water for injections. The solution was produced under GMP conditions.

### Stability Study

**Fig. 2:**
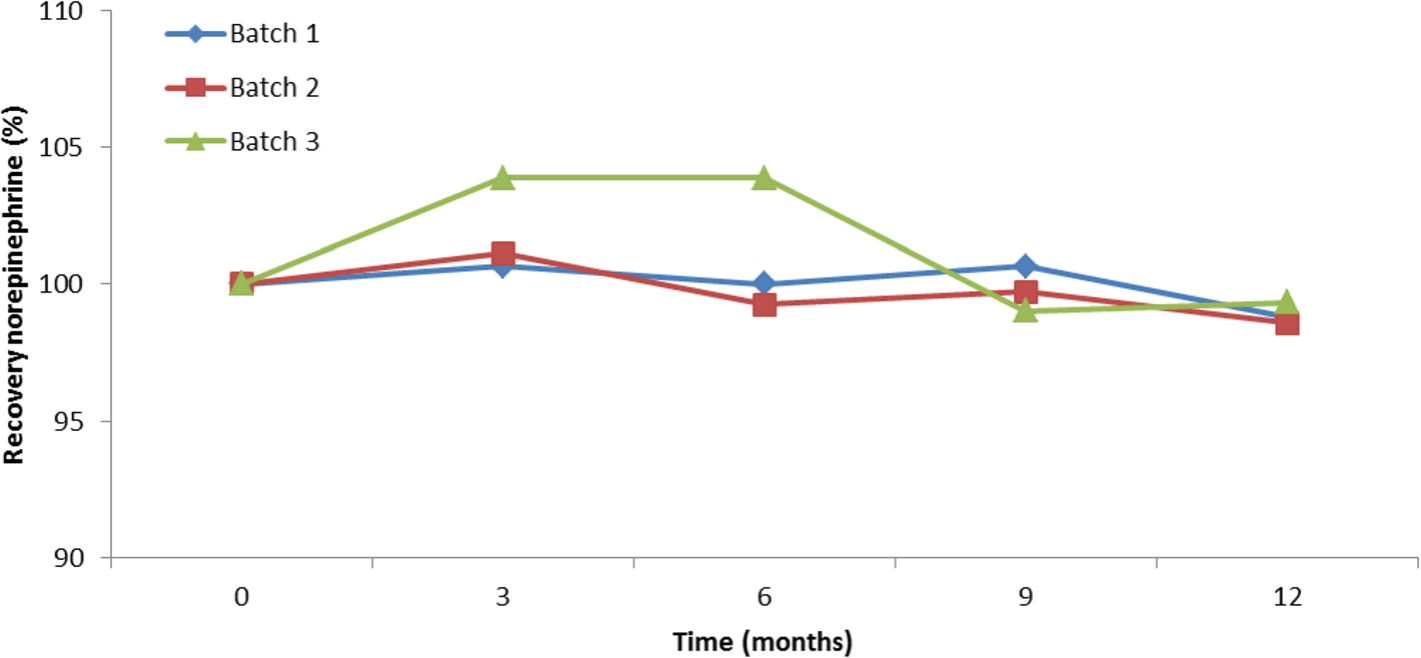
norepinephrine recovery (%) in stability study

The initial pH of 3.8–3.9 did not change significantly during storage; after 12 months, a pH of 3.8 to 4.0 was found. All solutions were clear at all points in time and the color of the solution was < B9, the solutions were sterile, and the number of particles was within limits. Table III gives an overview of all results at *t* = 12 months for the three batches. The norepinephrine recovery of the different batches is shown in Fig. 2. When analyzing the *t* = 0 content of the batches 1 and 2, it was found that there was less than 2% loss during production and sterilization. Based on this result, the overage of norepinephrine for batch 3 was reduced to 5%. According to the ICH guideline on pharmaceutical development, overages should be justified and proven safe (15). With our new approach, we were able to reduce the overage by 50% to only 5% instead of 10%, whereas even the old formulation containing 10% overage was safely used for decades. Moreover, our norepinephrine solution is administered to the patient with an infusion pump, where the dose will be titrated based on the clinical effect. So the solution can be labeled as a 0.10 mg/mL norepinephrine solution.

**Table III:** Results Stability Batches Norepinephrine Solution at *t* = 12 months

Test	Acceptance criteria	Batch 1	Batch 2	Batch 3
1. Clarity and degree of opalescence of the solution	Clear solution	Clear	Clear	Clear
2. Degree of coloration of the solution	≤ B9	B9	B9	B9
3. pH of the solution	3.6–6.0	3.8	3.8	3.8
4. Identity sodium	Positive	Positive	Positive	Positive
5. Concentration norepinephrine	95–105% at release; 90–110% end of shelf life.	98.8%	98.6%	99.3%
6. Concentration chloride	Concentration at release 95–105% Concentration end of shelf life 90–110%	100.6%	100.1%	101.5%
7. Subvisible particles	≤ 6000 particles/syringe for particles ≥ 10 μm ≤ 600 particles/syringe for particles ≥ 25 μm	1834 62	1160 44	3438 88
8. Closure integrity test	None of the vials contains any trace of blue-colored solution	No blue color	No blue color	No blue color
9. Sterility	Sterile	Sterile	Sterile	Sterile
10. Extractable volume	The volume extracted is not less than the nominal volume.	Complies	Complies	Complies

The recoveries of norepinephrine (concentration at *t* = 0 was set to 100%) were 98.8%, 98.6%, and 99.3% for batches 1, 2, and 3 respectively at *t* = 12 months.

Our study showed that a 0.1-mg/mL norepinephrine solution is stable without adding sodium metabisulfite as an antioxidant. The concentration of 0.1 mg/mL was chosen due to local use and the fact that the norepinephrine concentration does not affect the stability in a significant way (10). In the formulation study, ascorbic acid was shown to be unsuitable as an antioxidant for norepinephrine solutions. Ascorbic acid seems to have a destabilizing effect on norepinephrine based on the significantly lower concentration of norepinephrine found. Maybe it only has a synergistic stabilizing effect in combination with other preservatives (10). Further interference of peaks of the ascorbic acid and norepinephrine may have occurred. Since the solution without antioxidant showed adequate stability, it was decided to discontinue further analytical development on the solutions with antioxidant. This is in line with the results of Brustugun *et al*. (18) who found that bisulfite could have a destabilizing effect on epinephrine during exposure to light. A possible explanation could be the reaction of epinephrine with various free radicals. In addition, several studies have been performed in which dilutions of commercial products (including concentration sodium metabisulfite) were analyzed (19,20). Wolf *et al*. (19) show the stability of norepinephrine solution after dilution with 0.9% NaCl to 0.1 and 0.01 mg/mL throughout storage at a maximum of 8°C. Zenoni *et al*. (20) show stability up to 24 weeks for a 1:10 dilution of a commercially available 1.0-mg/mL norepinephrine solution at room temperature and at 2–8°C. Rather than sodium metabisulfite, other aspects of the formulation were probably having a more pronounced effect on stabilizing the solution. Palazzolo *et al*. (21) measured norepinephrine concentrations without adding any preserving additives, but the solutions were adjusted to three different pH values (1.96, 5.81, and 7.81) and stored at three different temperatures (− 60, 4, and 22°C). Both acidic and basic solutions were stable in the freezer, but at higher temperatures, degradation occurred fast. The stability of the solution is probably due to a synergistic effect of different stability parameters. The pH was set to 3.8–3.9, not only norepinephrine is more stable at acid conditions, but also the effect of sodium edetate is pH dependent (10,22).

Another advantage of removing sodium metabisulfite from the formulation is the fact that it can no longer cause unwanted side effects. Sulfite-containing medications may provoke adverse reactions with an immediate onset after injection (12,13). The signs and symptoms of sulfite hypersensitivity which may involve multiple target organs include bronchoconstriction, wheezing, dyspnea, laryngeal edema, swelling, and hypotension. Symptomology ranges in severity from mild discomfort to life-threatening episodes and even death (12). No accelerated stability study was performed, as this was not necessary as there was no time pressure on the development. And besides, norepinephrine solution is a well-known commercially available product (Levophed®, Arterenol®) with known degradation products (10). On the other hand, the shelf life study was carried out in real lifetime, with the shelf life claim never being longer than the demonstrated results.

Another challenge could be to demonstrate the closure integrity of the syringe. This was already performed in another study where the primary container in combination with the stopper and tip cap was investigated for suitability as primary packaging material for pharmaceutical products. For the closure integrity test in this study, positive control was included. This study shows all adequately closed syringes (17). Based on these results and the fact that the production process is similar for norepinephrine syringes, it was expected the norepinephrine syringes would also meet the acceptance criteria for closure integrity. This is confirmed in this study.

A weakness of the study could be that no impurities test has been performed. This must be taken into account when reviewing the results, although minimal effect is expected. For the production of the solutions, Ph. Eur. compliant raw materials were used which were within the acceptance limits at the release of the batch.

This is also the case for the determination of possible extractables from the primary container. This test is not repeated based on the results of an extensive qualification program performed for the primary container which show that the used cyclic olefin polymer syringes including stopper and tip cap are suitable as primary packaging materials for the production of water-soluble products with pH varying from 3 to 9 with having low extractable profile (17).

Our results with norepinephrine can be considered as an indication that also other catecholamines (like phenylephrine) can be formulated into ready-to-administer syringes without the use of sodium metabisulfite.

## CONCLUSION

The norepinephrine (0.1 mg/mL) solution containing sodium edetate and sodium chloride filled under nitrogen gassing in syringes followed by heat sterilization is stable for at least 12 months at room temperature when protected from daylight. Such a formulation can be free of antioxidants, like sodium metabisulfite or ascorbic acid.

## Abbreviations

COP: Cyclic olefin polymer
EDTA: Ethylenediamine tetraacetic acid
PFSS: Prefilled sterilized syringes
